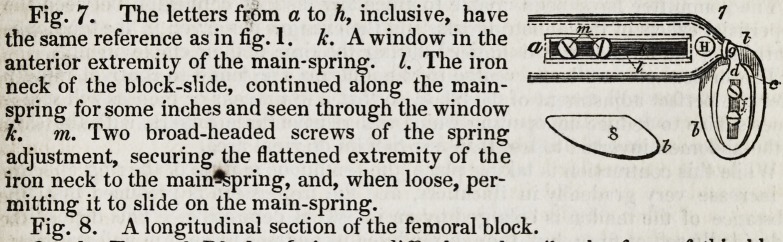# Report on the Radical Cure of Hernia by Means of Trusses with Solid Blocks, (Pads,)

**Published:** 1838-01

**Authors:** Reynell Coates, Isaac Parrish


### REPORT ON THE RADICAL CURE OF HERNIA BY MEANS OF TRUSSES WITH SOLID BLOCKS, (PADS.) READ BEFORE THE PHILADELPHIA MEDICAL SOCIETY, AND ORDERED TO BE PRINTED, APRIL 29TH, 1837.

BY REYNELL COATES, M.D., AND ISAAC PARRISH, M.D.

[In a former Number of this Journal, (Vol. III. p. 234,) we gave some account of the new method of treating hernia by means of trusses with solid wooden pads, introduced some years since in America. The present Report is the production of two experienced and eminent surgeons, and is founded on the continued observations and enquiries conducted by them since their appointment as a committee of examination in December, 1834. The practical results announced in it are of the highest importance; and, as they are based on actual observation, and are supported by the detail of cases, no doubt can be entertained of their authenticity; so that we seem, at last, justified in asserting that a radical cure for chronic hernia has been discovered in the American truss. The following extracts contain the more important facts in the Report. Of the six instruments invented by Dr. Chase we shall give figures and a detailed account of three only; but the principle and general plan of the whole will be sufficiently evident from our extracts.]

The trusses with solid blocks, now in use or recommended by inventors, may be divided into two classes. 1st. Those which are constructed for the express purpose of producing irritation, in order to effect a condensation of the skin, cellular tissue, and the fascia superficialis or the abdominal tendons about the hernial orifice, into one common mass by adhesion. 2d. Those which are designed to secure the constant, perfect, and safe retention of the bowel, without the attempt to create intentional irritation in the parts pressed by the instrument. The first class includes the truss of Stagner, and the various apparatus of Dr. Hood for the treatment of common inguinal, ventro-inguinal, femoral, and umbilical hernia.

The second class embraces all the instruments invented by Dr. Chase, which are five in number.

From the result of all the evidence presented to them, and their reasonings upon it, the committee are irresistibly drawn to the following conclusions. 1st. That the trusses of the first class do not secure the complete and permanent retention of the bowel with all the certainty which may be obtained by mechanical means. 2d. That although it is extremely probable that radical cures may be occasionally effected by the use of such instruments, it has not been proved that the success following their employment exceeds that which has been obtained by the better kinds of trusses previously in use. 3d. That the action of these instruments is often attended with serious and unnecessary inconvenience, uneasiness, and pain. Lastly, That their employment for too long a time, when the degree of pressure exerted by them is considerable, sometimes produces absorption of the tendons, dilatation of the hernial orifice, and an extension of the evils they are designed to remove; and that any attempt to obviate this danger, by lessening the pressure while the support of the instruments continues to be required, will diminish the security of the retention. For all which reasons the committee do not feel warranted in making a favorable report on the claims of this class of trusses upon the confidence of the society.

The object of the instruments of Dr. Chase is to secure the perfect and permanent retention of the viscera in hernia, in order to permit the powers of nature to effect a radical cure after the removal of the misplaced parts, which are supposed to offer the greatest obstacle to her success.

The inventions and improvements of Dr. Chase extend to all parts of the truss and its appendages, and his attention to minute but highly important details has been carried to an extent never equalled by any of his predecessors in this branch of surgery. The complete instruments employed by him are—1st. The Inguinal or Common Inguinal Truss. 2d. The Ventro-Inguinal Truss. 3d. The Femoral Truss. 4th. The Umbilical Truss. 5th. The Umbilical Belt. 6th. The Double Truss.

### I. *Of Inguinal or Common Inguinal Truss.*

Fig. 1. *a.* The extremity of the main-spring of the truss. *b.* The block. *c.* The brass block-rider: the screws by which it is attached being covered by the block-slide. *d.* The block-slide. *e.* The window in the block-slide. *f.* The two broad-headed screws of the block-adjustment, securing the rider to the slide, and, when loosened, sliding freely in the window. *g.* The soft iron flexible neck, attaching the block-slide to the main-spring. *h.* The button for the pelvic strap, which is generally used for the perineal strap also.

The proper perineal strap button on the end of the block-slide is omitted in this and some succeeding figures, to prevent confusion.

Fig. 2. Longitudinal section of the block.

Fig. 3. Transverse section of the same.

*Of the Block.* The block of this truss was warmly approved in the Preliminary

Report, and it has amply maintained its character throughout the more recent investigations: it is so perfectly adapted to the form of the parts interested in common inguinal hernia that the committee are unable to perceive in what manner it could be improved; nor has it ever failed, under their observation, in retaining the bowel both permanently and completely during the time of its employment, after the first few days required for the accurate adjustment of the instrument. Nothing farther appears necessary to prove the decided superiority of this block over all others known to the profession, in the particular form of hernia for which it is designed.

*Of the Block-attachment.* Two very important improvements upon the old modes of attaching the pad to the spring of the truss are observable in the block-attachment of the inguinal truss. The block is surmounted by a thin oval plate of brass, termed by the inventor a *block-rider;* and this is adapted to the under surface of an iron plate of nearly similar form, called the block-slide, to which it is attached by means of two round-headed screws, playing freely, when loosened a little, in a longitudinal fenestrum in the block-slide, so as to admit of any required change of the position of the block in this direction, to the extent of about an inch in the trusses designed for adults. The block-slide is connected to the spring by means of a round neck of soft iron, about three-quarters of an inch in length, sufficiently stiff to resist any change of shape during the most active movements of the patient, and sufficiently pliable to act like a universal joint under the hands of the surgeon. The combined action of the slide and the neck enables us to adjust the block with the utmost precision to the edge of Poupart's ligament, the rout of the abdominal canal, and the internal ring, whatever may be the peculiar form of the abdomen of the patient, while the block remains invariably in the exact position chosen by the surgeon; advantages possessed by none of the trusses previously in use, so far as they are known to the committee. These improvements are, in themselves, sufficient to add very greatly to the value of the instrument.

*Of the Spring and Strap attachment.* The endless varieties of form which have been given to the springs of trusses, render it apparently impossible that any thing intrinsically novel, in this part of the hernial apparatus, should be presented to the public hereafter; but it is of the utmost importance that the profession should determine what class of springs are calculated to give the greatest degree of security and permanency to the action of trusses.

This subject has been amply discussed in the work of Dr. Chase, and the committee are prepared, after due reflection, to coincide in the opinion expressed by that gentleman, that the semi-circular steel springs of Salmon and Ody are objectionable, because they are brought into accurate relation with the body only at the spots corresponding with the spine and the hernial orifice; the whole arch of the spring resting loosely over the side of the pelvis without a fixed location, and remaining liable to continual change of place from the movements of the glutei muscles and the reaction of the dress of the patient. The changes just mentioned must inevitably lead to the danger of corresponding changes in the position of the pads or blocks, and consequent insecurity of retention. The motives for the invention of this class of springs were the three following, and they are obviously fallacious. 1st. It was supposed that the pressure of the spiral elastic springs, being exerted throughout their whole length, renders them liable to derangement by the motions of the parts on which they press: but, excepting on the front of the hypogastric region of the abdomen, those parts have so slight a degree of mobility—based as they are upon the solid structure of the pelvis, and almost uninfluenced by muscular contractions, that their alterations of figure are of no real importance. The changes in the figure of the hypogastric region are fully compensated by the elasticity of the spiral springs, and those of the parts over the ring of the ilium are successfully counteracted by perineal straps, so that the accuracy and permanence of retention are not contravened when spiral springs are employed. 2d. It was supposed that the changes of shape in the hypogastric region required some mode of adjustment more complete than that effected by the elasticity of the main spring, to enable the pad or block to accommodate itself at all times to the form of the parts; and hence the ball-and-socket pad attachment, to which the semi-circular spring was deemed peculiarly adapted. But, if desirable, this mode of attachment may be as readily employed in connexion with the spiral spring. Your committee do not deem it desirable; because the ball-and-socket attachment renders secure but one point on the back of the pad or block, while the circumference may be tilted in any direction by the pressure of an intestine from within, almost as readily as by the movements of the abdomen, to which the pad is designed to yield; for the soft and compressible surface of the hypogastric region cannot securely prevent this tilting when the adjustment of the pad is not remarkably accurate, or when the propulsive force of the intestine in hernia is considerable. A third argument urged in favour of the introduction of semi-circular springs was drawn from the tendency of the strap attached to the spiral spring trusses to draw upwards, and thus displace the pad; but this difficulty is completely removable by giving to the spiral spring and the accessory parts of the truss a proper form and disposition, as will be explained hereafter.

Your committee are therefore of opinion that Dr. Chase has done wisely in adopting the spiral spring, and retaining the strap so as to encircle the whole pelvis by the truss, in preference to the semicircular spring and universal joint of Salmon and Ody's instrument, and the modifications of the same by the late Dr. Hull, of New York, the Rev. Mr. Reid, of Georgia, &c.

Although there is nothing positively novel in this part of the inguinal truss of Chase, the inventor has established definite rules for the degree of temper and the extent of the various curvatures of the spring, and also for the position of the strap-button, which render it easy to adjust the instrument more securely and permanently in all cases than can be done when these points are left to the discretion of instrument makers. Experience has decided that there is an advantage in giving an elastic temper to all that portion of the spring which intervenes between the pad-attachment in front and the opposite sacro-iliac symphysis in rear, but that the portion extending from the latter point to the opposite side of the pelvis should be so far softened as to admit of adjustment by being permanently bent. Three inches of the hinder extremity are left ductile in all the trusses of the full size; and thus the necessity of making an instrument expressly for each individual case (the great difficulty in the employment of spiral springs entirely of tempered steel,) is completely obviated, without sacrificing the accuracy of the adjustment on the one hand, or its permanency on the other.

It has been customary to curve downward the anterior end of the spiral spring, so that, when the part which lies across the back is horizontal, the front extremity may approach more nearly toward the abdominal canal. In Chase's inguinal truss this curvature does not exceed three-fourths of an inch, and its commencement is found far back upon the costa ilii when the instrument is applied; so that the spring, in passing forward from that point, winds downward below the anterior superior spinous process without encroaching too much upon the bellies of the glutii muscles or disturbing the proper position of the spring and strap on the back part of the pelvis. Any further increase of this curvature is attended with inconvenience, by giving the direction of the strap too much obliquity, and disposing the instrument to tilt upward in front; and such increase is rendered altogether unnecessary by the soft iron neck of the pad-attachment. In the last three inches of the anterior end of the spring there is another curvature, resulting from a slight tortion of the axis of the generating curve of the spring, which brings the flat side of this part of the spring into more complete correspondence with the surface of the hypogastric region, a matter of much importance to the comfort of the patient, and one giving additional security to the position of the instrument.

It has been customary, almost invariably, with truss-makers, to place the strap-button upon the plate or expansion which supports the pad; but Dr. Chase has very wisely affixed it to the anterior end of the spring: by which means the obliquity of the strap is much diminished, and the pelvis is enclosed by the instrument in a direction approaching very nearly to the circle, the strap lying altogether above the level of the block-slide, and the disposition of the instrument to tilt or ride upwards being reduced almost to nothing.

The committee consider the establishment of a fixed model for the triple curvature of the spiral spring, and the position of the strap-button, as a highly important recommendation to the instrument under notice.

*Of the Appendages.* The perineal strap is never wanting in the inguinal truss of Dr. Chase. It is attached behind by means of a sliding loop, through which pass the spring and cover. Before, it is commonly secured to the strap-button; but each instrument is also provided with another button, made expressly for the perineal strap. This is seated on the lower extremity of the block-slide, and may be used to give additional security and force to the action of the block when the lower part of the abdomen is very prominent and loaded with fat. The back-pad is a very important appendage to the truss, giving great certainty to the position of the instrument, by protecting from irritation the spinous processes of the sacrum, and filling the interval between the spring and the integuments along the median line on the back of the pelvis. Some very important improvements have been made in the construction and mode of attachment of this pad. It is formed of a simple circular disk of tin, about four inches in diameter, covered with soft buckskin, and lightly wadded. A broad sliding loop of leather suspends it on the spring and cover, so that its position may be adapted exactly to the size of the patient and other accidental circumstances. This perfectly free mobility of the back-pad is believed to be a novel arrangement, and one of high practical importance; for it is found that the parts about the back of the pelvis are so intolerant of even slight pressure, when very long continued, that the subcutaneous fat becomes absorbed and the skin irritated by the mildest back-pad, if it be worn in one invariable position for many months consecutively. This difficulty is entirely obviated by an occasional change of position, produced by sliding the pad a little toward one or the other side; a change that is not attended with any loss in the security of retention, and which is accomplished more readily by the arrangement just described than by any other known to the committee.

Having thus analysed the several parts of the inguinal truss of Dr. Chase, the committee feel bound honestly to state their conviction that this instrument surpasses all others known to them in the accuracy and permanency of its retentive power in common inguinal hernia; a conviction fully sustained by all their practical observations of the action of trusses. The instrument is worn with so much comfort, that patients generally relinquish it unwillingly, and sometimes ***absolutely refuse so to do,*** even when pronounced well by the surgeon.

The committee find themselves unable to suggest any improvement, or to point out any defect of principle or construction in this truss as now employed by the inventor.

II. *Of the Ventro-Inguinal Truss.*

Fig. 4. The attachment being in all respects similar to that in fig. 1, no references are required.

Fig. 5. Longitudinal section of the block.

Fig. 6. Transverse section.

The form of Chase's ventro-inguinal block is so accurately adapted to that of the os pubis, that it has secured the bowel perfectly in every instance of ventro-inguinal hernia in which it has been seen applied by the committee. The primary adjustment of the truss is considerably more difficult, and requires more time and skill in the worst cases of this accident than in the inguinal variety; but the ultimate success of retention does not appear to be less perfect when once accomplished. The pressure of this block upon the os pubis has been made a subject of complaint in only one instance, and the inconvenience then resulted from a slight maladjustment in the first application, which being corrected, the difficulty never recurred.

The only peculiarity of the Ventro-Inguinal Truss of Dr. Chase consists in the form of the block. In every other particular, it is identical with the inguinal truss. But, in the application of the instrument, it is necessary that the perineal strap should be secured, at its anterior extremity, to the button on the end of the block-slide, and not to that on the anterior extremity of the spring. To the complete instrument, as it has been actually employed during the last year, the committee may safely apply the same language used in concluding their remarks on the inguinal truss.

### III. *Of the Femoral Truss.*

Fig. 7. The letters from *a* to *h*, inclusive, have the same reference as in fig. 1. *k.* A window in the anterior extremity of the main-spring. *l.* The iron neck of the block-slide, continued along the main-spring for some inches and seen through the window *k.* *m.* Two broad-headed screws of the spring adjustment, securing the flattened extremity of the iron neck to the main-spring, and, when loose, permitting it to slide on the main-spring.

Fig. 8. A longitudinal section of the femoral block.

*Of the Femoral Block.* It is very difficult to describe the form of this block, and the committee will refer to the treatise of the inventor for the best description and an excellent woodcut representation of it. By considering the mechanical principles of its action, together with the only case fairly before the committee in which it has been employed, it is deemed safe to recommend it as preferable to any pad or block previously employed in this variety of hernia. It is calculated to preserve its position more accurately than the one before in use; it is not liable to become disturbed by the motions of the thigh; and it gives support in a direction which enables it to act at the greatest mechanical advantage. How far it may answer the special purpose of its construction, by entering under the fold of Poupart's ligament and acting almost directly on the femoral ring, the committee will not venture to judge upon the evidence of a single case. The report of Dr. Chase, as to its result in other instances, is favorable; but neither that gentleman nor the committee regard it as having acquired the highest degree of perfection of which it is capable. It will, probably, undergo further modification.

*Of the Block-attachment.* The extreme accuracy desirable in the adjustment of the small femoral block renders the mode of attachment a matter of great importance. Dr. Chase has succeeded in reaching, in this respect, a degree of perfection much higher than that attained by any of his predecessors. The relation of the femoral ring to the parietes of the pelvis varies in different individuals to a much greater extent than that of the abdominal canal, and its variations are not so nearly confined to one right line. The soft iron neck of the block-attachment in this truss is bent at a right angle, so as to place the long diameter of the block in a position perpendicular when the patient stands erect. In this position the motions of the block-slide, which are similar to those observed in the preceding trusses, adapt the block to the height of Poupart's ligament with great nicety; but to meet the peculiarities of individuals, in regard to the distance between the ring of the ilium and the femoral ring, another arrangement is necessary. There is a fenestrum, two inches in length, in the anterior extremity of the spring; and the soft iron neck, instead of being permanently secured to the spring, is elongated two or three inches, curved, flattened, and attached to the spring by means of two screws, which pass through the fenestrum, and, when loosened, play freely therein, so as to allow the block to approach or recede from the mesial line to any required degree. This double adjustment is simple, secure, and perfectly accurate.

There is no other peculiarity in the spring or appendages of this truss, but the perineal strap is always secured in front to the button on the bottom of the block-slide.

As regards the retentive power of the trusses which have been approved by the committee, it has been tested in various manners, and severely. Some of the patients, while wearing them, have followed the most trying labours of the harvest field and the marble-yard; others have travelled hundreds of miles on horseback, over mountainous countries. The subject of the worst incurable case of ventro-inguinal hernia, which had destroyed his usefulness, notwithstanding his endeavours to retain the bowel by means of other instruments, has since resumed his labours as a stevador and sailor; some have followed the chase, and leaped fences and dykes, gun in hand, &c.; yet, since the instruments were brought to their present high state of perfection, the committee know of no instance of protrusion under these exertions.

The committee have been unable to trace any distinct connexion between the superficial effects of these instruments, and the changes perceived in the tendinous margins of the hernial orifices noticed during the time of their employment.

The orifices of very large ventro-inguinal hernia are found to contract rapidly after the perfect adjustment of the block, so that a few weeks or months will sometimes suffice to reduce an opening which will receive three fingers, with the skin of the abdomen inverted to less than one half its original area.

While this contraction is taking place, the tendinous margin of the ring appears to increase very gradually in thickness, and the impression is produced that the substance of the tendon is enlarged by an intestinal deposition. This deposition is decidedly soft at first; but, though rendered by degrees more firm and resisting, it does not distinctly assume the well marked characters of the purely fibrous expansion in which it is formed, within any period yet determined, at least so far as can be ascertained by the sense of touch. In common inguinal hernia, even when they have become nearly direct, and in ventro-inguinal hernia of recent date, or moderate extent, the contraction and thickening continue on the increase until the affected ring is often rendered smaller, and, sometimes, much smaller than in the normal condition.

After all that has been stated, the committee feel themselves fully warranted in the following conclusions:

1. The retentive power of solid blocks is, *cæteris paribus*, superior to that of soft pads in the treatment of hernia.

2. The chances of radical cure depend upon the perfection and permanence of the retention.

3. The perfection and permanence of the retention depend—first, upon the mechanical action of the instruments; and, secondly, upon the power of the parts affected to bear that action without danger of physiological accidents of sufficient importance to interfere with the treatment.

4. All the instruments with solid blocks, contrived before the recent inventions of Dr. Chase, are decidedly liable to important mechanical objections.

6. The instruments of Dr. Chase have effected the permanent and accurate retention of the intestines in every case of hernia observed by the committee, without material inconvenience to the patient, and often under trials more severe than are usually ventured upon by those who wear other trusses; trials which would be imprudent with any other apparatus known to the committee.

7. If we except the Femoral Truss, these instruments have stood the test of much practical application without superinducing any physiological accidents of sufficient importance to interfere with the treatment.

8. The mechanical principles upon which the femoral truss is constructed appear highly ingenious and promising; and unless this instrument should be found hereafter to be productive of important physiological accidents, it must take precedence of all other modes of treating this variety of the disease. No such accidents are yet known to have been produced by its employment; but the committee have not enjoyed the opportunity of personal inspection in a sufficient number of cases to determine general results, nor do they deem it proper to receive evidence from any other quarter in discharging the trust reposed in them by the society.

The committee are induced by the foregoing conclusions to recommend, in strong terms, the instruments of Dr. Chase to the confidence of the profession, as the best known means of mechanical retention in hernia, and as furnishing the highest chances of radical cure.

All the individuals who have relinquished the use of the trusses approved by the committee, after having worn them for six months or more, and who have been afterwards examined by a member or members of the committee, have been subjected to the necessary tests, and are believed to be radically cured in the sense of the foregoing definition. A still larger number who are yet under treatment give promise of a similar result; and those who refuse finally to relinquish the instrument on the advice of their surgeon, present, in the firmness of the rings, and in the absence of protrusion under exertions performed when the trusses are temporarily removed, very strong grounds for believing the cure to be radical in them also.

The time required for the radical cure of an ordinary case of ventro-inguinal or direct hernia in the adult, appears to be from twelve to eighteen months. It is probable that the bowel, in common inguinal hernia, is rendered secure in a shorter time, but prudence has prevented the earlier relinquishment of the truss except in a very few cases. The orifice in umbilical hernia, appears to contract somewhat more slowly, but all the varieties recover much more rapidly in childhood.

*American Journal of Med. Sciences. Aug.* 1837.

---

IMPROVED FEMALE SYRINGE. BY HEBER CHASE, M.D.

## Figures and Tables

**Fig. 1 Fig. 2 Fig. 3 f1:**
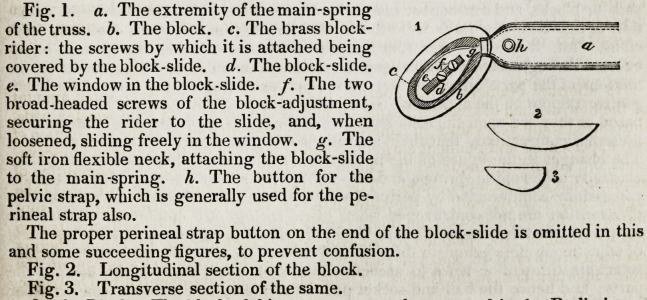


**Fig. 4 Fig. 5 Fig. 6 f2:**
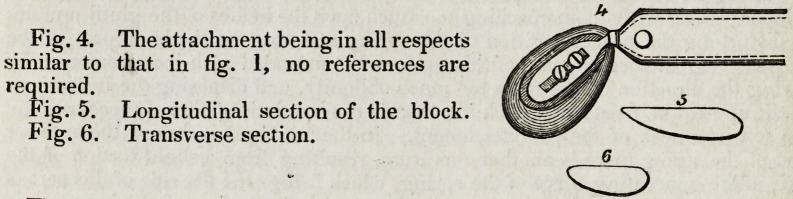


**Fig. 7 Fig. 8 f3:**